# Safe use of human milk for preterms in the context of maternal polypharmacy: a framework to improve practices

**DOI:** 10.1038/s41390-025-04416-z

**Published:** 2025-09-25

**Authors:** Dotan Shaniv, Anne Smits, Karel Allegaert

**Affiliations:** 1 School of Pharmacy, Faculty of Medicine, The Hebrew University of Jerusalem, Jerusalem, Israel; 2 Pharmacy Services, Kaplan Medical Center, Clalit Health Services, Rehovot, Israel; 3 Department of Neonatology, Kaplan Medical Center, Clalit Health Services, Rehovot, Israel; 4 Neonatal Intensive Care Unit, University Hospitals Leuven, Leuven, Belgium; 5 Department of Development and Regeneration, KU Leuven, Leuven, Belgium; 6 Department of Pharmaceutical and Pharmacological Sciences, KU Leuven, Leuven, Belgium; 7 Department of Hospital Pharmacy, Erasmus Medical Center, Rotterdam, The Netherlands; 8 Department of Neonatology, Hospital for Children and Adolescents, Leipzig University Medical Center, Leipzig, Germany; 9 Department of Paediatrics, University of Oxford, Oxford, UK; 10 Division of Neonatology, Department of Pediatrics, Acibadem Mehmet Ali Aydinlar University, İstanbul, Turkey; 11 Neonatal Intensive Care Unit, Fondazione IRCCS Ca’ Granda Ospedale Maggiore Policlinico, Milan, Italy; 12 Department of Paediatrics and Child Health, Infant Centre, University College Cork, Cork, Ireland; 13 Neonatal Intensive Care Unit, AOU Policlinico di Catania, Catania, Italy; 14 Neonatal Intensive Care Unit, Centre Hospitalier Intercommunal de Créteil, Créteil, France; 15 Department of Pediatrics, Clínica Universidad de Navarra, Madrid, Spain; 16 Division of Neonatology, Department of Pediatrics, Baskent University, Ankara, Turkey

## Abstract

**Abstract:**

Maternal pharmacotherapy during lactation is an ongoing research field, which relies on scientific evidence, pharmacological reasoning and extrapolation to support risk-to-benefit assessment. Typically, drugs are studied individually for potential effects on a nursing infant, with limited information on the preterm infant. Polypharmacy during lactation is still poorly studied, leaving healthcare professionals without any scientific guidance when consulting on human milk safety in this scenario. When focusing on a dyad of a postpartum mother on polypharmacy and a preterm infant, the benefits of mother’s own milk (MOM) should be weighed against the unknown potential risks of polypharmacy during lactation. Within this setting of limited evidence, a framework to improve clinical and research practices is provided. Possible measures to minimize the risk of maternal (poly)pharmacy to the newborn include: multi-disciplinary prenatal counseling; preferring medications with low passage rate into human milk; relative rating of medications according to safety profile; and ‘mixed feeding’ (alternating between MOM and other types of feed, preferably donor human milk). These measures can be used to support shared informed decisions on the risk-to-benefit assessment. As the topic of polypharmacy during lactation is still poorly explored, a research agenda is suggested.

**Impact:**

- Polypharmacy during lactation is poorly studied, and practical, evidence-based guidelines for healthcare professionals are lacking.
- Various methods can be employed to reduce exposure to medications through human milk, even more so when applied concomitantly: preference of medications with low passage into human milk, relative rating of medications according to known safety profile, or ‘mixed feeding’ where mother’s own milk is alternated with other types of feed, preferably donor human milk.
- A framework to improve clinical and research practices on provision of human milk in the setting of maternal polypharmacy and prematurity as an added challenge is provided.

## Introduction

### The importance of human milk (with focus on the preterm newborn)

Human milk is the recommended and preferred source of nutrition for newborns and young infants for its nutritional, immune and general health benefits. Human milk is known to reduce the risk for gastrointestinal diseases, of which the most prominent is necrotizing enterocolitis (NEC). Preterm infants receiving human milk may be 6- to 10-times less likely to develop NEC,^1,2^ while human milk components are being studied for their potential to mitigate various risk factors associated with NEC.^3^ This current view is also reflected in a joint consensus statement recently published by the U.S. Food and Drug Administration (FDA), the Centers for Disease Control and Prevention (CDC), and the National Institutes of Health (NIH).^4^ In addition, human milk supports normal development of the newborn microbiome and serves as a vital source of maternal antibodies during the initial postpartum period.^5^ Provision of human milk via direct breastfeeding is also known to confer physical and psychological health benefits for the mother, e.g., lower rates of metabolic disease conditions (hypertension, hyperlipidemia, type II diabetes, obesity) and cardiovascular diseases, lower rates of ovarian and breast cancer, lower risk of postpartum depression (PPD), increased chance for bonding with the infant, and more.^6–9^

The general recommendation is exclusive breastfeeding for the first 6 months of life,^10^ followed by weaning to a more varied diet that combines human milk and other food types (i.e., “partial breastfeeding”). This principle becomes particularly important in the case of preterm infants as they have higher nutritional requirements compared to term infants to “bridge the gap”,^11^ as well as a higher risk for NEC and other complications. There is evidence that human milk composition changes according to the level of prematurity as a biological attempt to meet the needs of the preterm infant since lipid, protein, and lactose levels have all been found to be consistently higher in milk produced following a preterm birth compared to term birth, for weeks and months postpartum.^12,13^ This highlights the importance of mother’s own milk (MOM) in this subgroup of infants, with donor human milk as first alternative. To further ensure that these higher nutritional requirements are met, the use of fortifiers is recommended.^14^

### Pharmacotherapy and polypharmacy during lactation

Maternal pharmacotherapy is highly prevalent postpartum, with a reported prevalence of postpartum medication use ranging from roughly one-third to almost all women.^15^ In a recent study that surveyed pregnant and lactating women across five European countries, breastfeeding women self-reported taking two concomitant medications on average^16^; this underscores the frequent occurrence of combination therapy (polypharmacy) in the postpartum period.

Notably, the majority of postpartum prescriptions are short-lasting treatments for acute conditions (e.g., analgesics and antibiotics), whereas a subset of mothers may require long-term maintenance pharmacotherapy for preexisting chronic diseases.^15^ Pregnancy complications may further increase postpartum medication burden: −44% of women with severe preeclampsia (often associated with preterm birth) required antihypertensive therapy after delivery, compared to ~1.8% among women without hypertensive disorders.^17^ Interestingly, 92.5%, 6.5%, 0.8% and 0.1% of the women used 1, 2, 3 and 4 medications, respectively.

**Fig. 1:**
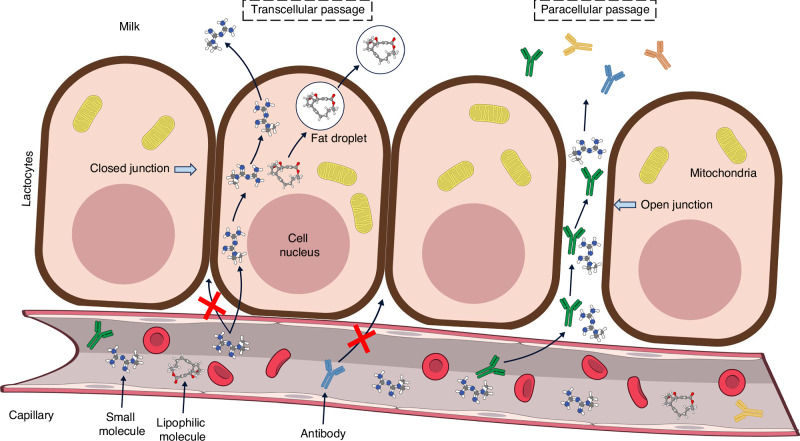
Pathways for drug transmission into human milk. ^19^ Open junctions are the gaps that exist between lactocytes during the first days postpartum and allow easier passage of small and large molecules. These junctions close after a few days, whereupon the passage of molecules is restricted to the transcellular passive diffusion pathway or specific facilitated transport mechanisms. See text. Image produced using NIAID Visual and Medical Arts^72^

It is currently well established that many medications may pass into human milk to some degree (Fig. 1). This knowledge is often a cause for concern for patients and healthcare providers who might be less familiar with lactational pharmacology. However, this research field is active and constantly developing, and many drugs have already been investigated for their safety profile during lactation, with data available in the medical literature.^18,19^ Where no such data are available, healthcare professionals often employ a set of widely accepted pharmacokinetic, pharmacological and physicochemical principles when consulting about pharmacotherapy during lactation. These principles have been extensively discussed elsewhere.^19–21^

The currently accepted position is that most studied drugs are not considered a contraindication for lactation.^19,22^ However, most of the available information on safety during lactation concerns exposure to a single medication through human milk, while data on drug combinations and the effect of possible drug interactions on the infant through human milk are very limited.

Therefore, deciding whether the human milk of a mother with polypharmacy may be given to the newborn is often difficult. Concerns for negative effects on the infant (particularly a preterm infant, in whom drug clearance capacity is low, such that accumulations and adverse effects are more likely to occur) may lead healthcare professionals to recommend against lactation with multiple drug therapy or offer inconsistent advice.^23,24^ On the other hand, a preterm newborn is expected to benefit significantly from human milk as mentioned above, to gain weight, receive maternal antibodies, or to mitigate the risk for NEC.

Potential exposure of a preterm infant to maternal polypharmacy through human milk warrants professional, case-by-case consultation, preferably by a multi-disciplinary team. However, when such consultation is not readily available, several principles may serve as an initial approach until an in-depth expert advice can be provided. The objective of this text is to present a framework of principles supporting the safe provision of MOM under maternal polypharmacy.

**Fig. 2:**
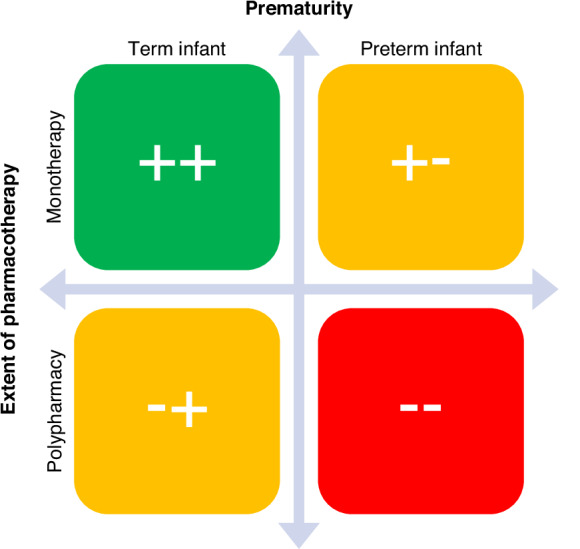
Extent of pharmacotherapy (single vs. multiple medications) and prematurity presented in a 2X2 matrix. The symbols schematically represent the potential complexity and level of uncertainty for decision-making under the given circumstances. While the combination in green is often simpler to evaluate (albeit not necessarily safe, as all decisions ultimately depend on the specific drug, the dose, available evidence, clinical reasoning and risk management), the combination highlighted in red has the highest perceived risk among these combinations.

The two primary determinants described herein are the extent of pharmacotherapy (maternal polypharmacy) and prematurity (similar exposure, lower drug clearance),^23^ which—when combined—form a spectrum of complexity for decision-making on the safety of pharmacotherapy during lactation (Fig. 2). While some of the presented principles apply to pharmacotherapy during lactation in general, others may apply specifically to the extreme case of polypharmacy and prematurity.

## Unique characteristics of preterm infants related to maternal drug therapy

### Prematurity

Prematurity is defined as birth before 37 + 0 weeks of gestation, and its severity is classified according to the gestational age (GA) at birth.^25,26^ There is an inverse relationship between GA and the overall condition of the preterm infant, i.e., the lower the GA at birth, the higher the general risk of the infant for severe neurodevelopmental outcomes and comorbidities of prematurity.^27^ Additionally, physiological differences between preterm and term infants affect the pharmacokinetics and -dynamics in the newborn^28^:

1. Immature digestive system in preterm infants is characterized by a relatively higher pH level in the stomach (relative achlorhydria), which gradually decreases as the newborn matures. This means that in preterm infants, oral absorption of acidic drugs will be decreased, while absorption of basic drugs may be increased compared to older children.
2. The total body water in extreme preterm infants is higher compared to late preterm and term cases (85-90% compared to 75% and lower, respectively).^29^ This means higher volume of distribution and lower plasma concentration for water-soluble drugs in the most immature preterm infants.
3. Immaturity of drug-eliminating organs (liver, kidneys) will lead to reduced hepatic metabolism and excretion processes of drugs in preterm infants compared to term infants. This may result in accumulation and higher drug exposure in the preterm infant compared to older infants.

Apart from the general immaturity that leads to pharmacokinetic differences in extremely preterm infants, the immaturity of the immune system, characterized by a lower number of immune cells and components such as monocytes, neutrophils and cytokines, makes preterm infants more vulnerable to environmental and maternal infections.^30^ For this reason, human milk that contains maternal antibodies is highly valuable, particularly during the first days postpartum (see below).

Another factor that should be taken into consideration is the required daily milk volume. Typically, daily drug exposure is normalized to infant bodyweight, i.e. the amount of drug per kg body weight per day. For preterm newborns, the absolute daily milk volume is lower than that of a term newborn or an older infant,. The volume of colostrum is limited (several tens of milliliters per day at most),^31^ while the commonly referenced volume of milk for an exclusively breastfed infant (whether preterm or term) in a later postnatal stage is approximately 150 mL/kg/day.^32^ Therefore, the younger the infant, he/she will receive lower total volume of milk. This would directly affect the absolute amount of drug(s) he/she will be exposed to through human milk, from which the daily drug exposure per kg of bodyweight is derived. However, the expected lower elimination capacity of a preterm infant should be taken into account when assessing exposure and the extent of potential effect.

Notably, if feeding strategy is “mixed”, i.e., alternating between MOM and donor milk/infant formula, the drug-containing milk volume the infant will receive will naturally be even lower. It should be noted that although such practice has never been specifically investigated for medicines, it may be considered as a viable option for limiting drug exposure through human milk based on straightforward pharmacokinetic reasoning. Moreover, such practice has been applied in cases where human milk was intentionally limited (e.g., for infants diagnosed with phenylketonuria, to reduce the amount of phenylalanine exposure),^33,34^ or unintentionally limited (i.e., when human milk production is insufficient for the needs of the infant).^35^ Thus, while not widely accepted as a method to reduce drug exposure through human milk, it is known to be applied in specific cases at the discretion of the mother and treating clinician.^36^

### Human milk

Human milk produced following a preterm birth is different in composition than milk produced following a term birth.^12,13^ Therefore, it should be considered that the higher fat percentage in the former may lead to higher concentrations of fat-soluble drugs in human milk (Fig. 1). Moreover, human milk following preterm delivery contains more immune elements (antibodies, anti-inflammatory components and immune cells) and a different microbiome compared to human milk following a term birth.^37^ During the first days postpartum, antibodies and large molecules can pass from the blood into the milk compartment through gaps between lactocytes (Fig. 1), which disappear during the first week of life, mainly driven by a decrease in progesterone levels.^38^ Therefore, during the first days postpartum, large molecules such as biological agents—otherwise considered safe during lactation—can pass into human milk.^19,39^

### Maternal postpartum condition

Sometimes, a mother that gives birth to a preterm infant has to remain hospitalized for an indefinite period of time due to her medical condition, e.g., (pre-)eclampsia, postpartum infection, prolonged pain; also, some mothers are receiving chronic drug therapy, such as psychotropic medications, diabetic medications, immunosuppressive drugs, drugs for cystic fibrosis, or biological agents etc., which they should continue in the postpartum period. Such medical conditions often require continuous drug therapy, and in certain cases, several concomitant medications. Such polypharmacy requires extra care in consultation due to potential synergistic effects, a large variety of possible drug combinations and lack of reliable data in the literature on their safety during lactation.

## Suggested considerations to support the provision of human milk to a preterm newborn at risk of exposure to maternal polypharmacy

Several considerations may support MOM provision (either through breastfeeding or administration of expressed milk) to a preterm whose mother receives several concomitant medications (subject to the safety profile of each drug, informed by commonly accepted sources^18,19^):

1. All newborns are expected to benefit greatly from human milk.^40,41^ In most cases, human milk exposure would outweigh the risk from exposure to drugs through human milk,^42^ as the latter is expected to be quite low (see below). Exceptions to this rule-of-thumb would be maternal high-risk infectious diseases (e.g., active, high viral load HIV, mastitis with Group B streptococcus (GBS), tuberculosis (TB) and more),^43^ mothers that actively use illicit drugs,^44^ mothers receiving certain types of chemotherapy or other compounds with known toxicity for the neonate, or neonatal conditions, e.g., infants diagnosed with phenylketonuria,^33,34^ galactosemia,^45^ or other conditions.^46^
2. A preterm newborn in the neonatal intensive care unit (NICU) is under constant observation through vital signs and clinical monitoring. Thus, in case one or more drugs to which they are exposed through human milk have any clinical effect on vital signs or other functions, it should be noticeable easily and quickly. It should be borne in mind that to identify such effects and trace them back to any medication the mother is taking, healthcare professionals must be familiar with possible effects of the maternal drugs on the infant and the available sources of information, like LactMed.^47,48^
3. There is a direct relationship between the time elapsed from birth and the volume of milk the infant requires. Older infants (e.g., 5–6 months old) may require higher absolute volumes of human milk than young neonates. Thus, during the first days after birth, when the newborn requires relatively small volumes of milk, the absolute amount of drug the infant may be exposed to is also expected to be relatively low. This may theoretically decrease the risk for clinical effects, even when the drug concentration in milk is thought to be relatively high. In the preterm, the lower absolute intake should be weighted to their immature metabolism and excretion processes. Furthermore, the preterm might also have been exposed to the drug(s) as a fetus, establishing exposure at delivery.

While these considerations comprise the general rule in favor of MOM, individual risk-to-benefit assessment should always be made, particularly at the lower end of the body weight spectrum.

**Fig. 3:**
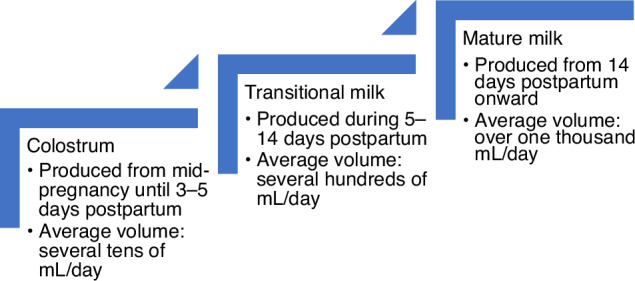
Development stages of human milk.^73,74^ The figure illustrates the transition from colostrum (produced in the first days after birth) to transitional milk, and subsequently to mature milk. Approximate durations and average daily volumes of each phase are shown.

When the mother receives several medications, some or even all may pose a potential risk to the newborn. The risk-to-benefit ratio of human milk provision should be thoroughly discussed (optimally, by a multi-disciplinary team and the mother) and an informed, shared decision should be made on therapeutic options, therapeutic plan, dosing and drug selection.^49,50^ As transitional milk replaces colostrum within 3–4 days from birth (Fig. 3), consumed milk volume is expected to increase rapidly,^51^ which in turn may require reassessment of overall drug exposure and safety of human milk exposure. In both term and preterm neonates, Yeung et al. suggested a ‘time window’ for high risk of exposure at 2–4 weeks of postnatal age, based on the weight-corrected milk volume intake and the overall still limited clearance capacity.^52^ Furthermore, this high-risk period is supported by epidemiological studies on adverse drug reactions in nursed infants.^53^ With similar weight-corrected milk volume intake in preterm to term infants, the preterm group is at risk for higher exposure and potential effects because of their lower clearance capacity.

## Recommendations to the healthcare providers treating the mother

As most literature on pharmacotherapy during lactation focuses on the objective safety profile of certain individual medications for the nursing infant, appropriate recommendations have been formulated to support provision of human milk in the challenging settings described above. These recommendations were initiated (June 2024) by a core team and adapted within the Paediatric and Neonatal Pharmacology Section of the European Society for Paediatric Research (ESPR). All contributors are listed in the acknowledgement section. In-person (October, 19, 2024) and online section meetings (November 20, 2024, March 17, 2025) and structured e-mail correspondence within the section were synthesized to reach agreement on the final version of these recommendations, also summarized in Table 1. In addition, published information from textbooks,^19^ professional societies communications^54^ and relevant journals^55^ or sources^18^ (including Pubmed database) were explored, and common medical and nutritional practices in the NICU, clinical and pharmacological reasoning and extrapolation concepts were hereby considered:

1. Drugs that are expected to achieve a low concentration in human milk should be preferred. This is a general recommendation that applies to all lactating women under drug therapy and therefore applies to mothers of preterm infants as well.
2. Where several therapeutic options are available for the condition of the mother, it is recommended to consult a pharmacist/clinical pharmacologist/teratology information specialist and review professional databases to choose the safest and most effective drug(s) and dosages within this framework.
3. In case the mother receives several drugs for the same indication (e.g., resistant hypertension, psychiatric illness requiring combination therapy), it is recommended to rate the drugs in relation to each other, based on the level of risk and extent of available safety information, and note this in the mother’s medical chart. Thus, whenever the treating physician will consider deprescribing, they may prioritize drugs considered to have the least favorable safety profile or those for which the least information is available, to minimize the exposure of the infant to these drugs and provide human milk as soon as possible.
4. In case the newborn is expected to be exposed to drugs for which no sufficient safety data in human milk is available, or that are known to pose a risk for the newborn, alternating between MOM and donor milk/infant formula to reduce exposure to those drugs while allowing the infant to receive some MOM can be considered. This risk management strategy aims to find the middle ground between potentially harmful drug exposure and the ever-so-important human milk. Although this practice has never been specifically and systematically investigated for medicines, it may be considered based on straightforward pharmacokinetic reasoning, similar to the general recommendations to avoid breastfeeding or to express human milk shortly after taking the drug when plasma concentration is building, i.e., within the “time to max” (*T*_max_), or taking a medication immediately after breastfeeding to allow drug levels to drop as much as possible (depending on drug half-life, this is mostly suitable for drugs with a relatively short half-life and short *T*_max_) before the infant has to feed again.^19,56,57^ This strategy may be applied to reduce exposure to drugs through human milk, as has been recommended for mothers with epilepsy taking medicines whose safety profile is yet undetermined.^58^ Such practice may also help to avoid sleep deprivation, which is known to lower seizure threshold.^59^

**Table 1:** Summary of recommendations for safe use of human milk in the context of maternal polypharmacy.

Recommendation	Reasoning
Prefer drugs expected to achieve low concentrations in milk.	This practice will ensure minimal infant exposure to drugs through milk, as exposure is a direct function of drug concentrations in milk.
Among similar therapeutic options, prefer drug(s) with the optimal safety profile for lactation according to specialized databases, clinical specialists and PK parameters.	Application of evidence-based knowledge will allow the clinician to choose the safest treatment option for the patient from a lactation point of view, while maintaining the quality of treatment for the mother herself.
Be aware of drugs’ safety profiles and rate them from best to worst. Make a note of this in the patient’s medical chart.	Awareness of the relative safety of several drugs prescribed for the same indication will allow the clinician to perform prudent deprescribing by discontinuing the least preferred drug(s) (from a lactation point of view) first.
When safety data is insufficient, consider reducing infant exposure by alternating MOM and donor milk (preferably) or infant formula.	Alternating between MOM and other types of feed reduces drug exposure while maintaining the benefits of human milk, at least in part. This strategy may be applied in any ratio that is believed to balance the benefits and risks. Even a small amount of MOM may have an impact on the infant as well as the mother. Over time, if MOM is well-tolerated, the ratio may be shifted in favor of MOM. As this strategy may be the most complicated to execute, its success may depend on thorough communication with the mother, shared decision-making, and adherence and compliance of the medical team.

*MOM* mother’s own milk, *PK* pharmacokinetic.

**Note**: This recommendation is particularly important during the first days postpartum when antibodies and other immune components may enter human milk, to allow the preterm newborn to receive at least some of them, but it may also be applied later to optimally balance the nutritional requirements of the infant and the potential risk from exposure to certain drugs.

It should also be noted that to maintain the benefits of human milk, the recommended order of priority is MOM > donor milk > infant formula.

## Common postpartum scenarios involving polypharmacy—why do these recommendations make sense?

To illustrate the rationale for the proposed recommendations, three arbitrarily selected common scenarios of postpartum polypharmacy were chosen: antihypertensive management for pre-eclampsia continuing into the postpartum period, antibiotic therapy for complicated postpartum infections and psychotropic medications for PPD. In each scenario it is demonstrated why, although not much evidence is available on polypharmacy, the theoretical risks may be minimized through the presented approach, to support prudent MOM provision with such drug regimen.

### Antihypertensive management for severe pre-eclampsia

Antihypertensives may be used in the postpartum period to treat chronic hypertension or a presentation of pre-eclampsia. Several therapeutic options are available from various drug classes, with some medications studied more than others during lactation. According to current data,^60^ hydrochlorothiazide and spironolactone (diuretics), nifedipine (a calcium channel blocker), enalapril (angiotensin-converting enzyme inhibitor), labetalol (beta-blocker) and hydralazine (vasodilator) are all considered safe during lactation, mainly because they are excreted into human milk in very low amounts (relative infant doses for all the above drugs are no more than 4.3%, while less than 10% is conventionally regarded as safe^19^). Thus, even if a postpartum woman would require several antihypertensives for controlling high blood pressure, choosing and combining from the abovementioned options (as well as other safe options not mentioned) should allow her to provide her own milk freely since exposure to the drugs through human milk is expected to be very low. As there is no information on cumulative exposure, it would be prudent to advise that relevant biomarkers (cardiovascular, renal, or other) of the newborn should be monitored (which is routinely done in the NICU anyway). This would virtually eliminate any serious drug reactions related to exposure to antihypertensives through human milk.

In the event that a less safe or less studied medication would be required as part of the maternal drug regimen (e.g., alpha-blockers, whose safety profile has not yet been sufficiently studied), the lowest effective dosage should be used. The treating doctor should take it into consideration that this is a less-favorable option from a lactation point of view and that it should be the first medication to be discontinued when possible. In certain cases, ‘mixed feeding’ may be considered (see above), when exposure may be predicted to be too high or too risky.

### Combination of antibiotic therapy postpartum for complicated infections

Postpartum infections usually require antibiotic treatment, and depending on the severity of infection, a combination of antibiotics may be required (e.g., for postpartum endometritis, septic shock, Methicillin-resistant Staphylococcus aureus infection, etc.). Many antibiotics are considered to be safe during lactation due to low passage into human milk,^61,62^ with common short-term risks being gastrointestinal adverse effects (vomiting, diarrhea, alteration of neonatal microbiome), fungal infections^63^ and giving the milk a foul taste,^19^ which may cause some discomfort to the feeding infant. Thus, when multiple antibiotics are prescribed for a postpartum woman, these risks may be cumulative and thus increased, and the infant should be monitored for them. However, this should not constitute a reason for interruption or discontinuation of MOM because these risks are usually minor and are not expected to have an overall significant clinical effect on the infant. Their presence would also be expected to be short-term, as most antibiotic treatments are usually limited to 5–10 days. If a significant gastrointestinal reaction is indeed witnessed, it may be advisable to have the mother temporarily express and discard her milk and the infant switched to donor milk/infant formula to avoid further exposure until the antibiotic treatment is over and provision of MOM may be resumed. Data on long-term outcome, e.g., impact on intestinal flora, need further exploration.

### Psychotropics for postpartum depression

Psychotropic medications are used for a variety of psychiatric indications, such as anxiety, psychosis, schizophrenia—all of which may occur in postpartum women, alongside the more specific indication for those patients, i.e., PPD.^64^ Pharmacotherapy for such indications may be either mono- or polytherapy, depending on the clinical state of the patient and illness history.

General considerations regarding psychotropic medication use during lactation include the treatment history of the patient (naïve or already under treatment); if treated during pregnancy and which treatment was prescribed (to avoid postpartum changes as much as possible); is there a caretaker that could help ease the burden of the mother, as breastfeeding can be demanding and sufficient maternal rest is necessary.^64,65^

Treatment modalities for PPD include mainly antidepressants, antipsychotics and mood stabilizers.^66^ Among those pharmacological groups, the preferred treatment options are those drugs whose passage into human milk is considered low. While benzodiazepines are better avoided during breastfeeding, short-term use of lorazepam and oxazepam is considered the least risky due to poor passage into human milk, relatively short half-lives and lack of active metabolites. For the antidepressants, sertraline is considered a preferred compound due to low passage into milk, and quetiapine is a preferred antipsychotic, although other similarly safe options are available from both groups.^67,68^ Most psychotropic medications have been reported to possibly increase the risk of drowsiness in the infant, which may lead to feeding difficulties, failure to gain weight, etc.^69^

Although hardly any evidence exists, it may be reasonable to assume that this risk of drowsiness may be cumulative and thus may be increased when several culprit medications are taken by the mother. Thus, a newborn receiving MOM of a mother who is taking such medications should be clinically monitored by the mother herself, as well as by her close network or a healthcare professional, particularly during the first days postpartum. In specific cases, where the maternal psychiatric condition may be adversely affected by the overwhelming life changes brought about by the newborn and their care, the wellbeing of the mother may be maintained and the risks mitigated by employing “mixed feeding”, i.e., formula/bottle feeding at night by partner/another caretaker, to allow the mother sufficient rest.

In summary, if a postpartum mother is adequately controlled with a combination of psychotropic medications with low passage rates into human milk and a known optimal safety profile, MOM provision can be recommended, subject to regular infant monitoring, both in and out of hospital, and maintaining of maternal well-being as much as possible. It is generally suggested that MOM provision with an underlying maternal psychiatric disease should always be based on a case-by-case evaluation by a multi-disciplinary medical team.

## Suggested research agenda

Most available information on the safety of pharmacotherapy during lactation is based on single medication exposure. Such reports can describe adverse effects (or lack thereof) with a certain level of confidence, as there is no concern for confounding by multiple drug exposure. Consequently, no guidelines are available for polypharmacy during lactation, and consultations on such cases are often accompanied by a caveat of “the effects of multiple drug exposure during lactation on the infant are largely unknown”.

Being aware that broader consultation is warranted, a proposed research agenda is hereby described as a call to illuminate this not uncommon question of the safety of polypharmacy during lactation. The suggested research agenda has been constructed by applying the methodological approach described earlier:

- Elucidating common drug combinations during lactation through pharmacoepidemiological research can help focus efforts on the safety of such combinations during lactation. Which drugs are usually administered simultaneously to a postpartum mother? Some common combinations may include analgesics for uncontrolled postpartum pain, antibiotics for postpartum infections, antidepressants/anxiolytics/mood stabilizers (usually as a continuation of a chronic, prepartum treatment regimen).
- What possible mechanisms may complicate drug exposure due to polypharmacy during lactation compared to monotherapy? What types of drug interactions may occur, and how can they affect infant exposure? Would plasma or milk drug concentrations be affected by polypharmacy, in the sense of pharmacokinetic interactions such as competitive binding to transporters in lactocytes,^70^ or changes in milk pH which may alter the ability of an ionizable drug molecule to pass into milk?
- Regarding drugs for chronic conditions that may be used prenatally, perinatally and postnatally, is there any difference in effects in the infant between postnatal-only exposure (i.e., by lactation only) and exposure during lactation when preceded by intrauterine exposure? May intrauterine drug exposure be a protective factor against adverse drug effects (e.g., due to earlier exposure and/or attainment of a steady state in the fetus), or could it make the infant more prone to adverse drug effects? To answer this question, comparative observational case-control or cohort studies may be required.
- How can the effects (i.e., pharmacodynamics) of polypharmacy during lactation be quantified in the infant? How would the risk-to-benefit ratio be measured? Possible solutions may include physiologically-based pharmacokinetic modeling (PBPK)^71^ or targeted literature search on clinical case reports where polypharmacy is described in detail. PBPK models hold the promise to quantify drug uptake in nursing infants, driven by maternal and infant physiology, as well as drug properties.
- Is it possible to devise an evidence-based approach to mixed feeding composed of MOM and donor human milk (preferably) or infant formula in ratios that would balance the benefits of human milk, the nutritional requirements of the infant and minimal drug exposure?
- What are the current practices used, resources and supportive care available in different NICUs? As no specific and evidence-based guidelines are available, it might be interesting to study how polypharmacy during lactation is handled in various NICUs around the world. Surveys assessing local practices and points of view may be helpful in assessing the level of attention this area receives. Sharing and comparing views and opinions may help to reach some agreement among experts, which may form a basis for clinical guidelines and recommendations.

## Summary

Human milk is the recommended source of nutrition for newborns, and preterm infants in particular. If the mother is receiving pharmacotherapy with multiple drugs, her milk may contain various drugs in variable concentrations, but this should not be considered a contraindication for the provision of MOM, as certain strategies may be employed to mitigate potential risks. While optimal guidance regarding the safety of lactation under drug therapy (especially polypharmacy) can only be provided on a case-by-case basis, the principles described above may facilitate the shared decision-making process and make decisions more robust. Such an approach may help optimize the rates of newborns receiving MOM. We also suggest putting the research agenda provided herein into action to expand the existing knowledge base in this setting.
